# Spleen participation in partial MHC class II construct neuroprotection in stroke

**DOI:** 10.1111/cns.13369

**Published:** 2020-03-31

**Authors:** John Brown, Chase Kingsbury, Jea‐Young Lee, Arthur A. Vandenbark, Roberto Meza‐Romero, Halina Offner, Cesar V. Borlongan

**Affiliations:** ^1^ Department of Neurosurgery and Brain Repair Center of Excellence for Aging and Brain Repair University of South Florida College of Medicine Tampa FL USA; ^2^ Neuroimmunology Research R&D‐31 VA Portland Health Care System Portland OR USA; ^3^ Department of Neurology and Molecular Microbiology & Immunology Oregon Health & Science University Portland OR USA

**Keywords:** cerebral ischaemia, cytokines, immune response, major histocompatibility complex class II, middle cerebral artery occlusion, regenerative medicine, spleen

## Abstract

Pathological progression of stroke in the peripheral and central nervous systems (PNS and CNS) is characterized by multiple converging signalling pathways that exacerbate neuroinflammation‐mediated secondary cell death. This creates a need for a novel type of immunotherapy capable of simultaneously lowering the synergistic inflammatory responses in the PNS and CNS, specifically the spleen and brain. Previously, we demonstrated that partial major histocompatibility complex (MHC) class II constructs can be administered subcutaneously to promote histological and behavioural effects that alleviate common symptoms found in a murine model of transient stroke. This MHC class II manipulates T cell cytokine expression in both PNS and CNS, resulting in dampened inflammation. In our long‐standing efforts towards translational research, we recently demonstrated that a potent next generation mouse‐based partial MHC class II construct named DRmQ (DRa1_L50Q_‐mMOG‐35‐55) similarly induces neuroprotection in stroke rats, replicating the therapeutic effects of the human homolog as DRhQ (DRa1_L50Q_‐human (h)MOG‐35‐55) in stroke mice. Our preclinical studies showed that DRmQ reduces motor deficits, infarct volume and peri‐infarct cell loss by targeting inflammation in this second species. Moreover, we provided mechanistic support in both animal studies that partial MHC class II constructs effectively modulate the spleen, an organ which plays a critical role in modulating secondary cell death. Together, these preclinical studies satisfy testing the constructs in two stroke models, which is a major criterion of the Stroke Therapy Academic Industry Roundtable (STAIR) criteria and a key step in effectively translating this drug to the clinic. Additional translational studies, including dose‐response and larger animal models may be warranted to bring MHC class II constructs closer to the clinic.

## 1 INTRODUCTION

Cerebral ischaemia is a leading cause of death in the world and remains the primary cause of long‐term disability in the United States.^1^, ^2^, ^3^ Ischaemic stroke accounts for 87% of stroke incidents, with nearly one‐third of the patients succumbing to death, while another 20% to 30% becoming severely and permanently disabled. ^1^, ^2^, ^3^ Ischaemic stroke is characterized by an acute primary cell death response, followed by secondary cell death in the subacute and chronic phase.^1^, ^2^, ^3^ Several signalling pathways accompany secondary cell death in stroke, including exacerbated inflammation in both the central and peripheral nervous system (CNS and PNS). This inflammation is considered a primary culprit in secondary cell death.^4^

Effective treatment of ischaemic stroke relies on a narrow timeline that disqualifies more than 90% of patients.^5^, ^6^, ^7^ Patients face a limited number of therapeutic options: tissue plasminogen activator (tPA), mechanical reperfusion, stroke unit care and rehabilitation.^5^, ^6^, ^7^, ^8^ Furthermore, for tPA, the current standard of care, treatment of ischaemic stroke must commence within 4.5 hours.^9^ Therefore, novel approaches that extend the therapeutic window of tPA are warranted.^8^, ^9^, ^10^ To this end, the subacute and chronic evolution of inflammation presents an opportunity to develop innovative stroke therapies.^8^, ^11^

Perturbed immune response represents a key underlying pathology of many neurovascular, autoimmune and traumatic injuries of the CNS including stroke. Secondary cell death involves multiple pathways, but a major process entails a feedback loop of neuroinflammation, resulting in hypoxic tissues, vascular damage and blood‐brain barrier (BBB) leakage, expanding outward from the region of damage.^3^, ^4^, ^5^ The aberrant immune response‐associated secondary cell death in stroke manifests as an initial acute inflammatory response characterized by influx across the BBB of activated mononuclear cells subsequently evolving into chronic and often progressive deterioration of the neurovascular unit. Neutrophils stand as key cellular mediators of the immune response, acting as the first immune cell‐type recruited to the ischaemic area.^12^ During stroke progression, T cells are also activated,^13^ serving as a major inflammatory trigger to BBB extravasation.^14^ Accordingly, targeting any of these immune and inflammatory cells may attenuate the secondary cell death associated with stroke.^15^, ^16^, ^17^, ^18^, ^19^ While promising laboratory studies demonstrate effective pharmacological sequestration of stroke inflammatory cell death by mitigating intrusion of immune and inflammatory cells into the ischaemic penumbra during the acute phase, there exists no robust treatments against the progressive inflammation in the chronic phase. Partial MHC II constructs take advantage of the interplay between central and peripheral inflammatory responses, in which the spleen plays a critical role in modulating secondary cell death by manipulating T cell cytokine expression in both splenic and neurovascular inflammatory pathways.^20^, ^21^, ^22^, ^23^, ^24^, ^25^ In this review, we discuss preclinical evidence demonstrating that partial MHC II constructs pose as potent stroke therapeutics. In particular, that these constructs target both central and peripheral immune systems represents an innovative treatment strategy for stroke. The translational research challenges that warrant further investigations to bring partial MHC II constructs to clinical applications are also presented. The ultimate goal of this review paper is to offer a critical assessment of existing discovery‐ and mechanism‐based data and identify gaps in knowledge towards the advancement of safe and effective partial MHC II constructs as stroke therapeutics.

## 2 PARTIAL MHC II CONSTRUCTS SEQUESTER NEUROINFLAMMATION IN ANIMAL MODELS OF CNS DISORDERS

To address this gap in inflammation‐targeted treatment, a series of laboratory investigations examined the efficacy of partial major histocompatibility complex (MHC) class II construct DRa1‐mMOG‐35‐55 (DRa1 domain covalently linked to mouse (m)MOG‐35‐55 peptide), which is an immune‐targeted therapeutic that significantly antagonizes the acute recruitment and activation of brain‐infiltrating T cells caused by CNS insults.^11^, ^20^ These partial MHC class II constructs successfully reduced inflammation, infarct volume and cognitive deficits in animal models of four separate neurodegenerative and neuroinflammatory CNS conditions, including experimental autoimmune encephalomyelitis (EAE), methamphetamine addiction, traumatic brain injury and ischaemic injury.^11^

Encouraged by positive efficacy readouts in animal models of neurological disorders, a second more potent generation of partial MHC II constructs, namely DRhQ (DRa1_L50Q_‐human (h)MOG‐35‐55), has been developed for clinical development^11^, ^21^ and DRmQ (DRa1_L50Q_‐mMOG‐35‐55) for preclinical testing.^21^ DRa1_L50Q_‐mMOG‐35‐55 is effective in treating EAE in mice, a model of multiple sclerosis.^21^ In parallel, the DRmQ precursor, DRa1‐mMOG‐35‐55, reduces stroke symptoms in middle cerebral artery occlusion (MCAo) stroke mice^22^ and in distal middle artery occlusion dMCAO stroke mice by shifting microglia/macrophages towards the antiinflammatory M2 phenotype.^23^ In EAE, DRmQ increases the binding affinity for the CD74 receptor, thereby enhancing the ability of DRa1‐mMOG‐35‐55 to competitively inhibit both macrophage migration inhibitory factor (MIF) and D‐dopachrome tautomerase (D‐DT) from binding CD74 and delivering downstream inflammatory effects.^21^, ^22^, ^24^ CD74, MIF and D‐DT are commonly elevated after CNS conditions, such as stroke and EAE.^24^ Since DRhQ is the human homolog of DRmQ, it can circumvent the need for class II tissue typing due to its conserved DRα1 moiety, making it ideal for translation to the clinic.^11^

Following thromboembolic stroke, there is a significant increase in activated monocytes and neutrophils in the ischaemic cortex, as well as early activated T cells in the spleen.^20^ Previous studies in mice with stroke as well as traumatic brain injury have shown that partial MHC II inhibitors provide neuroprotection by significantly attenuating this aberrant immune response in both the central and peripheral nervous system.^11^, ^20^ We advanced the mechanisms by which DRmQ targets both central and peripheral inflammatory responses, including the attenuation of splenic inflammation, as effective means of conferring stroke neuroprotection.^11^, ^26^ Our recent study on DRmQ^26^ represents the first time that this promising therapeutic has been tested in a rat stroke model. Here, we review the preclinical evidence providing the basic science rationale and translational approach in nurturing the entry of partial MHC II constructs from the laboratory to clinical application.

## 3 TESTING DIFFERENT PARTIAL MHC II CONSTRUCTS: FROM DRα1‐hMOG‐35‐55 (DRhQ) To DRa1_L50Q_‐mMOG‐35‐55 (DRmQ)

**Figure 1:**
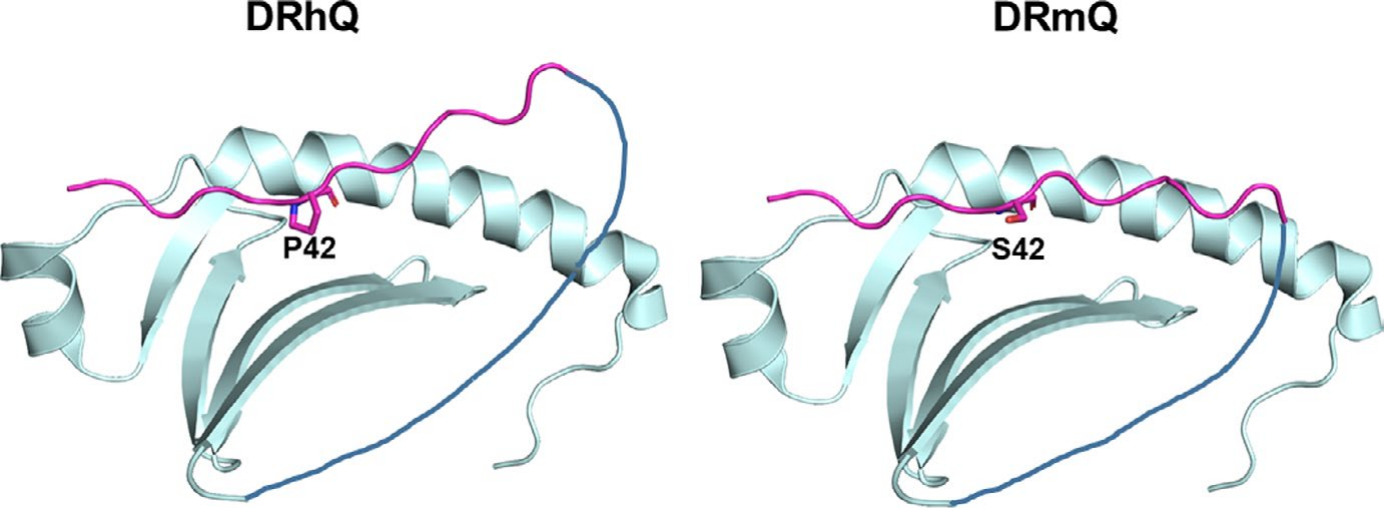
Partial MHC II Contructs, DRmQ and DRhQ. DR refers to one of the three human leucocyte antigen (HLA) isotypes along with DP and DQ. The Q refers to a glutamine for lysine amino acid substitution at residue 18 of the DRα1 domain in both constructs. The “h” and “m” refer to the origin of the antigenic MOG‐35‐55 peptide: “h” from human MOG and “m” from mouse origin. The C terminus of the antigenic peptide (in magenta) is covalently linked to the N‐terminus of the DRα1 domain through a flexible peptide linker (black). The human MOG peptide has a proline (P) at position 42, whereas the mouse peptide has a serine (S) at the same position. Structurally, in the DRhQ molecule, the antigenic peptide is bent due mostly to 2 adjoining proline residues. This might explain the difference in activity of the two constructs

The human homolog of partial MHC II construct is called DRhQ, while the mouse homolog is DRmQ (Figure 1). DRhQ demonstrated treatment efficacy by reducing neuroinflammation in multiple animal models of neurological disorders, including stroke and traumatic brain injury.^11^ Partial MHC II constructs work as recombinant T cell receptor ligands to competitively bind with CD74 in order to prevent it from binding with MIF, thus significantly reducing acute immune inflammation response following CNS conditions such as EAE.^22^ Both the MHC and peptide components on these human recombinant T cell receptor ligands (RTLs) are required for treatment efficacy of EAE.^11^ Partial MHC II constructs inhibit acute immune cell entry from the peripheral nervous system in stroke mice, and subsequently help promote polarization towards an M2 antiinflammation phenotype in the CNS.^8^ Building on this novel therapeutic pathway, a new generation of the compound named DRmQ (DRa1_L50Q_‐mMOG‐35‐55) was created, in which a single amino acid substitution (L50Q) in the DRα1 domain strengthens its binding affinity for CD74, and thus enhancing its antiinflammatory properties.^21^ Strong competitive inhibition is critical, since studies have shown in mice that stroke induces swift and massive activation of the PNS immune system that subsequently influences and harms the CNS.^20^ DRmQ and DRhQ effectively treat an EAE mouse model by binding to the cell surface receptor, CD74, that putatively blocks the binding of two inflammatory factors, MIF and D‐DT.^21^ Because of the robust inflammatory response seen in stroke secondary cell death, we embarked on testing the partial MHC II constructs in the middle cerebral artery occlusion (MCAo) models, initially testing Dra1‐mMOG‐35‐55 in the stroke mouse,^8^, ^10^, ^20^, ^22^, ^23^, ^24^, ^25^ and recently evaluating DRmQ in the stroke rat^26^ (Table 1). We focus the subsequent sections to DRmQ in our efforts to satisfy translational enabling criteria towards clinical application of partial MHC II constructs in stroke.

**Table 1:** Milestone stroke studies utilizing DRhQ and DRmQ

Partial MHC II inhibitor	Study	Animal	Significance
DRα1‐MOG‐35‐55 (HLA‐DRα1 domain linked to MOG‐35‐55 peptide)	MCAO Stroke (Benedek et al,^22^ 2014)	Mice	Inhibited neuroantigen‐specific T cells Demonstrated binding to CD‐74 and inhibiting MIF. Reduced symptoms incurred from stroke. Reversed splenic atrophy after stroke
DRα1‐MOG‐35‐55 (HLA‐DRα1 domain linked to MOG‐35‐55 peptide)	dMCAO Stroke (Wang et al^23^, 2017)	Mice	Inhibited neuroantigen‐specific T cells Demonstrated binding to CD‐74 and inhibiting MIF. Reduced symptoms incurred from stroke.
DRα1‐MOG‐35‐55	Traumatic Brain Injury (Yang et al^24^, 2017)	Mice	Significantly reduced CNS inflammation and improved clinical and histological results after traumatic brain injury
DRmQ and DRhQ (DRa1L50Q‐mMOG‐35‐55) and (DRa1L50Q‐hMOG‐35‐55) More potent versions of partial MHC II constructs DRα1‐MOG‐35‐55 with single amino acid substitution (L50Q) in the DRα1 domain	EAE (Meza‐Romero et al^21^, 2019)	Mice	DRmQ and DRhQ provide stronger binding affinity for CD‐74 receptor. Better efficacy than DRα1‐MOG‐35‐55 due to greater inhibition of D‐DT and MIF from binding to CD74 receptor.
DRmQ (DRa1L50Q‐mMOG‐35‐55) A more potent version of partial MHC II constructs DRα1‐MOG‐35‐55 with single amino acid substitution (L50Q) in the DRα1 domain	MCAO Stroke (Lee et al,^26^ 2019)	Rats	Provides a second animal model utilizing the potent next generation partial MHC II construct inhibitor This helps fulfil translation into clinic

## 4 DRmQ TARGETS THE SPLEEN IN AMELIORATING IMMUNE RESPONSE IN STROKE MODELS

The PNS and CNS share neuroinflammatory pathways; therefore, it is critical to address both areas of inflammation.^20^, ^26^ Poststroke inflammation includes a rapid activation of microglia followed by the infiltration of peripheral inflammatory cells, including neutrophils, T cells, B cells and macrophages.^11^ The spleen harbours a number of immune cells, and in response to injury upregulates cytokines in the blood and subsequently the brain after stroke.^27^ In a rodent MCAo model of stroke, splenocytes are detected in the injured hemisphere of the brain at 48 and 96 hours.^28^ Removing the spleen in mice before stroke significantly reduces lesions, activated macrophages, microglia and neutrophils in the brain after stroke, thereby further reinforcing the presence of significant sharing and crosstalk between the PNS and CNS inflammatory pathways.^18^, ^29^, ^30^, ^31^, ^32^, ^33^, ^34^ Splenectomy may be particularly problematic for older patients where immune cells demonstrate a propensity to work aberrantly and disrupt critical homoeostatic pathways related to brain regeneration and repair.^35^, ^36^ That splenectomy may not work for aged patients solicits finding innovative pharmacologic approaches that equally sequester inflammation, which may prove more tolerable and effective than a surgical manoeuvre.

Treatment with DRmQ appears to achieve the therapeutic effects of splenectomy in that DRmQ reduced the proinflammatory cytokines interleukin 6 and tumour necrosis factor (TNF)‐α in the spleen.^26^ Moreover, DRmQ treated rats exhibit significantly increased spleen weights.^26^ This supports the hypothesis that DRmQ attenuates the inflammation response in both the PNS and CNS.^26^ More importantly, it demonstrates that attenuation of inflammation in the spleen is critical to the pathology and treatment of stroke. Preventing splenic atrophy following stroke has been shown to attenuate inflammation and damage in the central nervous system due to the spleen releasing fewer immune cells to the pathway of the brain.^37^ This also implies that DRmQ may be useful in chronic treatment of stroke, in concert with other strategies designed to sequester the deleterious splenic immune response.^38^, ^39^ That DRmQ affords both CNS and PNS antiinflammatory effect is uniquely important, because if a partial MHC II construct treats the brain, but has no effect on the spleen, then the inflammation incurred by the spleen may still cause significant damage.^8^, ^11^ DRmQ’s increased binding affinity for CD74 makes it vitally more potent in attenuating both CNS and PNS immune system.^11^, ^40^ Additionally, while our studies focused on the spleen, DRmQ may exert additional peripheral effects that ameliorate stroke‐induced impairments beyond dampening the spleen inflammatory response. Indeed, targeting the MHC may affect stem cells mobilizing them into the circulation^41^ or enhancing their differentiation and altering the gut microbiome,^42^ altogether potentially fostering therapeutic effects on stroke.

## 5 DRmQ DAMPENS NEUROINFLAMMATION IN THE STROKE BRAIN

The expression of Iba1‐activated microglia and proinflammatory cytokine TNF‐α are endemic to neuroinflammation in the CNS following cerebral ischaemia and may cause further damage when left untreated.^39^ DRa1‐mMOG‐35‐55 has been also shown to exert similar therapeutic benefits in the distal MCAo stroke model characterized by reducing infarct size, modulating microglia polarization towards antiinflammatory phenotype and decreasing proinflammatory cytokines such as IL‐1α and IL‐17.^8^, ^10^, ^11^ Microglia, which normally serve as the brain's local macrophages, may differentiate into a proinflammatory M1‐like phenotype or antiinflammatory M2‐like phenotype.^8^, ^40^ However, it should be noted these distinct M1‐versus‐M2 processes have been recently disputed and are more intricately viewed on a spectrum rather than a binary classification.^43^, ^44^, ^45^

Middle cerebral artery occlusion stroke rats treated with DRmQ display significantly reduced expression of TNF‐α cytokines and ionized calcium binding adaptor molecule‐1 (Iba1)‐activated microglia and macrophages.^26^ This may explain the significant attenuation of secondary inflammation, reduction of infarct size, and increased cell survival demonstrated in this study.^18^ MCAo stroke rats treated with DRmQ also exhibit significantly improved motor and neurological performances.^26^

As noted above, our preclinical studies implicate the potential of DRmQ to exert therapeutic effects in both the CNS and the PNS, but acknowledged that with construct's enhanced BBB penetration, CNS may be preferentially targeted. In determining the envisioned clinical dosing regimen, optimizing the timeline of drug administration and exploring the possibilities to advance DRmQ either as stand‐alone or adjunctive treatment to tPA are warranted. Evaluating its synergistic treatment with tPA and potentially expanding the therapeutic time window are interesting future translational studies.

## 6 THE FUTURE OF PARTIAL MHC CLASS II CONSTRUCTS: ENABLING STUDIES FOR TRANSLATION FROM THE LABORATORY TO THE CLINIC

**Figure 2:**
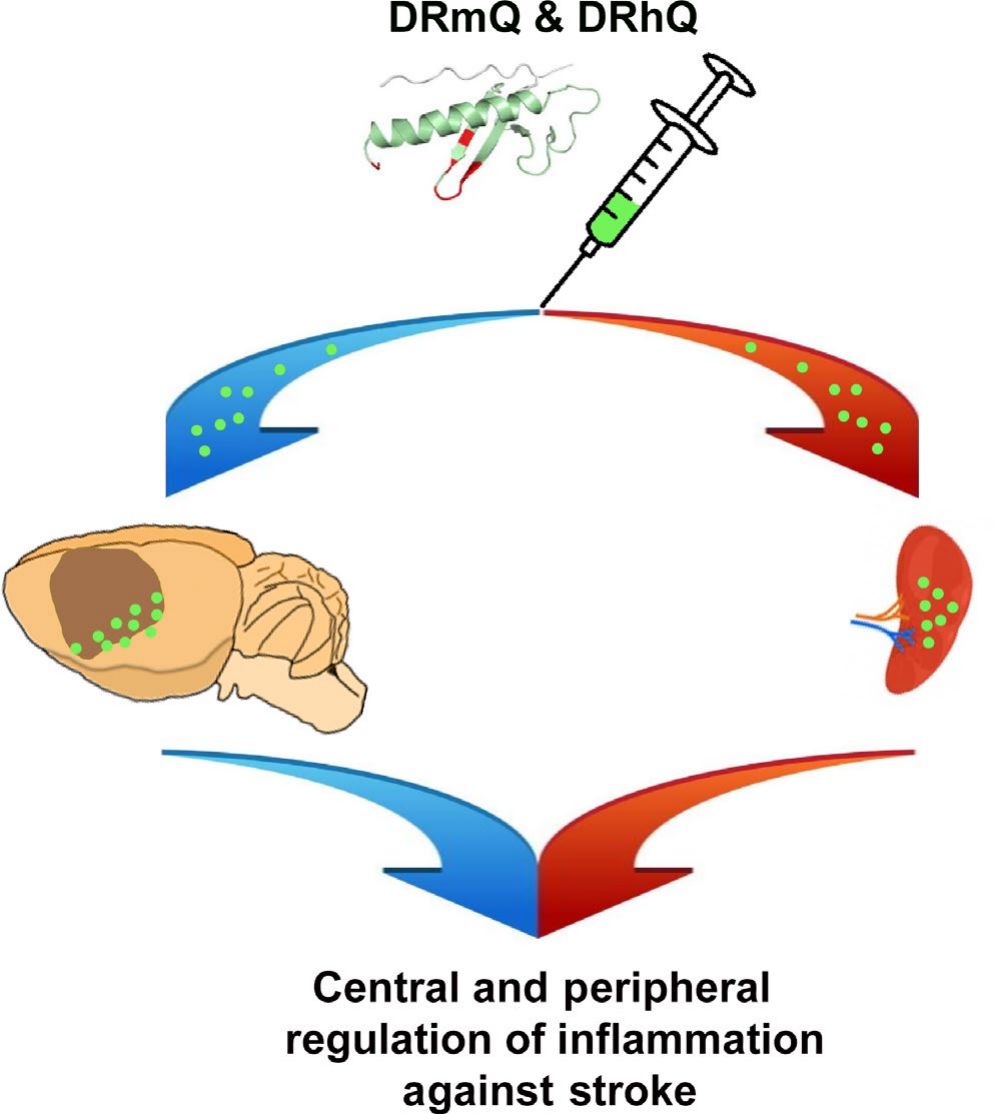
Therapeutic effects of DRmQ in stroke. Subcutaneous DRmQ alleviates stroke‐induced behavioural deficits, reduces cerebral infarct volume and peri‐infarct cell loss, lowers stroke‐induced inflammatory response in the brain and attenuates the splenic inflammatory response

DRmQ, DRhQ and other partial MHC II constructs have the potential to provide much needed alternative drug treatments that may extend the therapeutic window for ischaemic stroke, traumatic brain injury and similar neurodegenerative effects^11^, ^25^, ^26^ (Figure 2). The Stroke Therapy Academic Industry Roundtable (STAIR) criteria have been instituted to effectively translate this drug into the clinic.^9^ At least two different species of stroke model are necessary to fully assess the clinical relevance of partial MHC II constructs. The highlighted study represents the first treatment with partial MHC II construct DRmQ on rats for ischaemic stroke.^26^ This is now the second animal species in which DRmQ has demonstrated support for treatment efficacy. Previous studies provided evidence of DRmQ treatment efficacy in mice.^11^, ^21^ Partial MHC II constructs such as DRhQ do not require tissue typing due to the conserved DRα1 moiety of the drug shared between DRmQ and its human homolog DRhQ.^11^, ^21^ This MHC‐independent effect of partial MHC II constructs in conjunction with favourable preclinical treatment results in multiple CNS conditions in a variety of species should hasten bringing DRhQ to clinical trials.^21^, ^26^

Our preclinical studies on DRhQ and DRmQ based on animal models of stroke provided solid evidence of safety and efficacy of these compounds in treating stoke, but more mechanism‐based and translational studies are warranted.^26^ While many neurological disorders are considered as “brain” pathological disorders, there is also a major peripheral component.^8^, ^26^, ^36^, ^37^ This would entail that a holistic approach addressing both the brain and periphery that will improve therapeutic outcomes, as opposed to merely targeting the brain. This would also support the notion that treating the periphery may promote robust central effects. With these principles in mind, treating splenic inflammation may prove to dampen neuroinflammation, since many of the circulating inflammatory cells and molecules originate from the spleen, and may likely be sequestered at that level, thereby reducing their influx into the brain.^38^ Utilizing partial MHC II constructs may also provide much needed synergistic effects with current treatments such as tPA, by adding an immune‐targeted therapeutic modality to attenuate the acute proinflammatory response incurred from cerebral ischaemia.^8^

It will be essential to perform dose‐finding studies to identify the optimal dosage and potential side effects, as well different routes of administration and therapeutic window.^3^, ^36^ Furthermore, a longitudinal study will be helpful in ascertaining whether DRmQ has treatment benefits extending into the chronic phase of stroke. DRmQ appears to already have a longer time frame of use than current stroke therapies, but a longitudinal study is still needed to see how long that time frame may be extended.^11^, ^26^ We did not detect any significant harmful histological or behavioural side effects, but studies extending into the chronic phase may elucidate detrimental mechanisms or possibly even better therapeutic results.^26^ Since DRmQ was demonstrated to target the spleen, a study focused on infusing it directly into the spleen may yield beneficial results. Comparing DRmQ treated stroke rats with or without a splenectomy may further illuminate how much DRmQ attenuates the peripheral nervous system's immune response and whether manipulation of splenic T cells is a key mechanism to its attenuation. Splenectomy prior to injury has been shown to reduce neuroinflammation and provide neuroprotection in various brain injury models including stroke.^40^ Lastly, it may be helpful to pair DRmQ with other therapies known to decrease immune inflammation in the spleen and CNS. To this end, stem cell treatment by administration to the spleen has been shown to help reduce the immune system's inflammatory response.^38^, ^40^ Treatment with human bone marrow‐derived mesenchymal stem cells that demonstrate homing and antiinflammatory effects promoted a 40 per cent downregulation of TNF‐α in the spleen.^40^ Similar antiinflammatory approaches have been tried in stroke models.^46^, ^47^, ^48^, ^49^, ^50^ Partial MHC II constructs either as stand‐alone or in combination with these antiinflammatory treatments may prove as robust stroke therapeutics.

The immune disturbance in stroke has been recognized as a major exacerbating factor in the secondary cell death in stroke. While the secondary cell death entails multiple degenerative processes, the aberrant immune and inflammatory response appears to produce the most devastating compromise to the BBB leading to ischaemic penumbra expansion^3^, ^4^, ^5^ The onset of the destructive proinflammatory response is detected as early as a few hours of stroke episode and remains upregulated even months after stroke onset, thereby providing a wider therapeutic window to test promising treatments. Recognizing the main cellular mediators of the immune response will be key in identifying potential therapeutic; for example, neutrophils are the earliest immune cell‐type recruited to an ischaemic injury.^12^ Over time, stroke induces prolonged T cell activation,^13^ which are cells that primarily contribute to inflammatory extravasation of the BBB.^14^ Targeting any of these immune and inflammatory mediators may be therapeutic for stroke. Unfortunately, to date, no effective treatments have been found in the clinic to sequester these stroke immune and inflammatory responses. Partial MHC II constructs specifically target these immune and inflammatory responses, both centrally and peripherally, thus they stand as potent stroke therapeutics.

## 7 CONCLUSION

Partial MHC II constructs are effective as stroke therapeutics. In addition to the CNS, DRmQ targets the PNS, in particular the spleen, in attenuating peripheral inflammation, as well as preventing weight loss of the spleen, which is typically incurred after stroke.^26^, ^37^ Additionally, DRmQ is effective in both stroke mice and rats, thereby fulfilling a critical STAIR criterion of demonstrating efficacy in a second species.^9^ Large animal models, such as gyrencephalic primates or cats, may be desirable additional second species for preclinical stroke therapeutic testing and may be warranted as the next critical step if additional translational questions cannot be answered in the rodent models. Other potential translational hurdles as we bring these compounds to the clinic include the need for longitudinal studies that extend into the chronic stage of stroke, as well as the incorporation of dosage and therapeutic window optimization, which will further build on the safety and efficacy profile of the DRmQ. The unique mechanistic action of partial MHC II constructs on the immune system may work synergistically with other stroke treatments,^8^, ^49^ such as tPA, thus advancing their use either as stand‐alone or adjunct therapeutics.

## CONFLICT OF INTERESTS

Drs. Offner, Vandenbark, Meza‐Romero and OHSU have a significant financial interest in Artielle ImmunoTherapeutics, Inc, a company that may have a commercial interest in the results of this research and technology. This potential conflict of interest has been reviewed and managed by the OHSU and VA Portland Health Care System Conflict of Interest in Research Committees. All other authors declare no conflict of interests.

## References

[1] Nishino H , Borlongan CV . Restoration of function by neural transplantation in the ischemic brain. Prog Brain Res. 2000;127:461‐476. 11142041 10.1016/s0079-6123(00)27022-2

[2] Hara K , Yasuhara T , Maki M et al Neural progenitor NT2N cell lines from teratocarcinoma for transplantation therapy in stroke. Prog Neurogibol. 2008;85:318‐334. 10.1016/j.pneurobio.2008.04.005 18514379

[3] Borlongan CV . Cell therapy for stroke: remaining issues to address before embarking on clinical trials. Stroke. 2009;40:S146‐S148. 19064801 10.1161/STROKEAHA.108.533091 PMC4810678

[4] Blecharz‐Lang KG , Wagner J , Fries A et al Interleukin 6‐mediated endothelial barrier disturbances can be attenuated by blockade of the IL6 receptor expressed in brain microvascular endothelial cells. Transl Stroke Res. 2018;9:631‐642. 29429002 10.1007/s12975-018-0614-2

[5] Simon R , Meller R , Yang T , Pearson A , Wilson G . Enhancing base excision repair of mitochondrial DNA to reduce ischemic injury following reperfusion. Transl Stroke Res. 2018;10:664‐671. 30535792 10.1007/s12975-018-0680-5 PMC6842339

[6] Navarro‐Oviedo M , Roncal C , Salicio A et al MMP10 promotes efficient thrombolysis after ischemic stroke in mice with induced diabetes. Transl Stroke Res. 2018;10:389‐401. 30051168 10.1007/s12975-018-0652-9

[7] Griemert EV , Recarte Pelz K , Engelhard K , Schäfer MK , Thal SC . PAI‐1 but not PAI‐2 gene deficiency attenuates ischemic brain injury after experimental stroke. Transl Stroke Res. 2018;10:372‐380. 29978354 10.1007/s12975-018-0644-9 PMC6647425

[8] Benedek G , Vandenbark AA , Alkayed NJ , Offner H . Partial MHC class II constructs as novel immunomodulatory therapy for stroke. Neurochem Int. 2017;107:138‐147. 27773790 10.1016/j.neuint.2016.10.007 PMC5411346

[9] Fisher M , Feuerstein G , Howells DW , et al. Update of the stroke therapy academic industry roundtable preclinical recommendations. Stroke. 2009;40:2244‐2250. 19246690 10.1161/STROKEAHA.108.541128 PMC2888275

[10] Zhu W , Casper A , Libal NL et al Preclinical evaluation of recombinant T cell receptor ligand RTL1000 as a therapeutic agent in ischemic stroke. Transl Stroke Res. 2015;6:60‐68. 25270354 10.1007/s12975-014-0373-7 PMC4298461

[11] Vandenbark AA , Meza‐Romero R , Benedek G , Offner H . A novel neurotherapeutic for multiple sclerosis, ischemic injury, methamphetamine addiction, and traumatic brain injury. J Neuroinflammation. 2019;16:14. 30683115 10.1186/s12974-018-1393-0 PMC6346590

[12] Yao H‐W , Kuan C‐Y . Early neutrophil infiltration is critical for inflammation‐sensitized hypoxic‐ischemic brain injury in newborns. J Cereb Blood Flow Metab. 2019:271678X19891839 10.1177/0271678X19891839. [Epub ahead of print]. PMC7585929 31842667

[13] Xie L , Li W , Hersh J , Liu R , Yang SH . Experimental ischemic stroke induces long‐term T cell activation in the brain. J Cereb Blood Flow Metab. 2019;39:2268‐2276. 30092705 10.1177/0271678X18792372 PMC6827125

[14] Steiner O , Coisne C , Engelhardt B , Lyck R . Comparison of immortalized bEnd5 and primary mouse brain microvascular endothelial cells as in vitro blood‐brain barrier models for the study of T cell extravasation. J Cereb Blood Flow Metab. 2011;31:315‐327. 20606687 10.1038/jcbfm.2010.96 PMC3049495

[15] Benedek G , Chaudhary P , Meza‐Romero R et al Sex‐dependent treatment of chronic EAE with partial MHC class II constructs. J Neuroinflammation. 2017;14:100. 28477623 10.1186/s12974-017-0873-y PMC5420407

[16] Meza‐Romero R , Benedek G , Yu X et al HLA‐DRα1 constructs block CD74 expression and MIF effects in experimental autoimmune encephalomyelitis. J Immunol. 2014;192:4164‐4173. 24683185 10.4049/jimmunol.1303118 PMC4028955

[17] Zarriello S , Neal EG , Kaneko Y , Borlongan CV . T‐regulatory cells confer increased myelination and stem cell activity after stroke‐induced white matter injury. J Clin Med. 2019;8(4):537. 10.3390/jcm8040537 PMC6518209 31010132

[18] Xu K , Lee JY , Kaneko Y et al Human stem cells transplanted into the rat stroke brain migrate to the spleen via lymphatic and inflammation pathways. Haematologica. 2019;104:1062‐1073. 30514806 10.3324/haematol.2018.206581 PMC6518907

[19] Neal EG , Acosta SA , Kaneko Y , Ji X , Borlongan CV . Regulatory T‐cells within bone marrow‐derived stem cells actively confer immunomodulatory and neuroprotective effects against stroke. J Cereb Blood Flow Metab. 2019;39:1750‐1758. 29569981 10.1177/0271678X18766172 PMC6727132

[20] Dotson AL , Chen Y , Zhu W , Libal N , Alkayed NJ , Offner H . Partial MHC constructs treat thromboembolic ischemic stroke characterized by early immune expansion. Transl Stroke Res. 2016;7:70‐78. 26627498 10.1007/s12975-015-0436-4 PMC4828931

[21] Meza‐Romero R , Benedek G , Gerstner G et al Increased CD74 binding and EAE treatment efficacy of a modified DRα1 molecular construct. Metab Brain Dis. 2019;34:153‐164. 30353480 10.1007/s11011-018-0331-2 PMC6364671

[22] Benedek G , Zhu W , Libal N , Casper A , Yu X , Meza‐Romero R . A novel HLA‐DRα1‐MOG‐35‐55 construct treats experimental stroke. Metab Brain Dis. 2014;29:37‐45. 24122483 10.1007/s11011-013-9440-0 PMC3975671

[23] Wang J , Ye Q , Xu J et al DRα1‐MOG‐35‐55 reduces permanent ischemic brain injury. Transl Stroke Res. 2017;8:284‐293. 27988839 10.1007/s12975-016-0514-2 PMC5418106

[24] Yang L , Liu Z , Ren H et al DRα1‐MOG‐35‐55 treatment reduces lesion volumes and improves neurological deficits after traumatic brain injury. Metab Brain Dis. 2017;32:1395‐1402. 28303450 10.1007/s11011-017-9991-6 PMC5600636

[25] Zhu W , Libal NL , Casper A , Bodhankar S , Offner H , Alkayed NJ . 301 Recombinant T cell receptor ligand treatment improves neurological outcome in the presence of tissue plasminogen activator in experimental ischemic stroke. Transl Stroke Res. 2014;5:612‐617. 24953050 10.1007/s12975-014-0348-8 PMC4125526

[26] Lee JY , Castelli V , Bonsack B et al. A novel partial MHC Class II construct, DRmQ, inhibits central and peripheral inflammatory responses to promote neuroprotection in experimental stroke. Transl Stroke Res. 2019. [Epub ahead of print]. 10.1007/s12975-019-00756-1 PMC10166182 31797249

[27] Offner H , Subramanian S , Parker SM , Afentoulis ME , Vandenbark AA , Hurn PD . Experimental stroke induces massive, rapid activation of the peripheral immune system. J Cereb Blood Flow Metab. 2006;26:654‐665. 16121126 10.1038/sj.jcbfm.9600217

[28] Seifert HA , Hall AA , Chapman CB et al A Transient decrease in spleen size following stroke corresponds to splenocyte release into systemic circulation. J Neuroimmune Pharmacol. 2012;7:1017‐1024. 23054371 10.1007/s11481-012-9406-8 PMC3518577

[29] Esposito E , Ahn BJ , Shi J et al Brain‐to‐cervical lymph node signaling after stroke. Nat Commun. 2019;10:5306. 31757960 10.1038/s41467-019-13324-w PMC6876639

[30] Lee JY , Acosta S , Tuazon JP et al Human parthenogenetic neural stem cell grafts promote multiple regenerative processes in a traumatic brain injury model. Theranostics. 2019;9:1029‐1046. 30867814 10.7150/thno.29868 PMC6401413

[31] Lee JY , Lin R , Nguyen H et al Central and peripheral secondary cell death processes after transient global ischemia in nonhuman primate cerebellum and heart. Methods Mol Biol. 2019;1919:215‐225. 30656633 10.1007/978-1-4939-9007-8_17

[32] Acosta SA , Mashkouri S , Nwokoye D , Lee JY , Borlongan CV . Chronic inflammation and apoptosis propagate in ischemic cerebellum and heart of non‐human primates. Oncotarget. 2017;8:102820‐102834. 29262526 10.18632/oncotarget.18330 PMC5732692

[33] Gonzales‐Portillo C , Ishikawa H , Shinozuka K , Tajiri N , Kaneko Y , Borlongan CV . Stroke and cardiac cell death: two peas in a pod. Clin Neurol Neurosurg. 2016;142:145‐147. 26866777 10.1016/j.clineuro.2016.01.001

[34] Ishikawa H , Tajiri N , Vasconcellos J et al Ischemic stroke brain sends indirect cell death signals to the heart. Stroke. 2013;44:3175‐3182. 24008571 10.1161/STROKEAHA.113.001714 PMC3859251

[35] Nguyen H , Zarriello S , Coats A et al Stem cell therapy for neurological disorders: a focus on aging. Neurobiol Dis. 2019;126:85‐104. 30219376 10.1016/j.nbd.2018.09.011 PMC6650276

[36] Stonesifer C , Corey S , Ghanekar S , Diamandis Z , Acosta SA , Borlongan CV . Stem cell therapy for abrogating stroke‐induced neuroinflammation and relevant secondary cell death mechanisms. Prog Neurogibol. 2017;158:94‐131. 10.1016/j.pneurobio.2017.07.004 PMC5671910 28743464

[37] Seifert HA , Offner H . The splenic response to stroke: from rodents to stroke subjects. J Neuroinflammation. 2018;15:195. 29970193 10.1186/s12974-018-1239-9 PMC6030736

[38] Acosta S , Tajiri N . Intravenous bone marrow stem cell grafts preferentially migrate to spleen and abrogate chronic inflammation in stroke. Stroke. 2015;46:2616‐2627. 26219646 10.1161/STROKEAHA.115.009854 PMC4542567

[39] Acosta SA , Lee JY , Nguyen H , Kaneko Y , Borlongan CV . Endothelial progenitor cells modulate inflammation‐associated stroke vasculome. Stem Cell Rev Rep. 2019;15:256‐275. 30739275 10.1007/s12015-019-9873-x PMC6441406

[40] Wang Z , He D , Zeng Y et al The spleen may be an important target of stem cell therapy for stroke. J Neuroinflammation. 2019;16:20. 30700305 10.1186/s12974-019-1400-0 PMC6352449

[41] Finkielsztein A , Schlinker AC , Zhang L , Miller WM , Datta SK . Human megakaryocyte progenitors derived from hematopoietic stem cells of normal individuals are MHC class II‐expressing professional APC that enhance Th17 and Th1/Th17 responses. Immunol Lett. 2015;163:84‐95. 25454068 10.1016/j.imlet.2014.11.013 PMC4278953

[42] Biton M , Haber AL , Rogel N et al T Helper cell cytokines modulate intestinal stem cell renewal and differentiation. Cell. 2018;175(1307–1320):e22. 10.1016/j.cell.2018.10.008 PMC6239889 30392957

[43] Rosi S . A polarizing view on posttraumatic brain injury inflammatory response. Brain Circ. 2016;2:126‐128. 30276287 10.4103/2394-8108.192517 PMC6126276

[44] Liu ZJ , Ran YY , Qie SY et al Melatonin protects against ischemic stroke by modulating microglia/macrophage polarization toward anti‐inflammatory phenotype through STAT3 pathway. CNS Neurosci Ther. 2019;25:1353‐1362. 31793209 10.1111/cns.13261 PMC6887673

[45] Kerr N , Dietrich DW , Bramlett HM , Raval AP . Sexually dimorphic microglia and ischemic stroke. CNS Neurosci Ther. 2019;25:1308‐1317. 31747126 10.1111/cns.13267 PMC6887716

[46] Zhang W , Zhao J , Wang R et al Macrophages reprogram after ischemic stroke and promote efferocytosis and inflammation resolution in the mouse brain. CNS Neurosci Ther. 2019;25:1329‐1342. 31697040 10.1111/cns.13256 PMC6887920

[47] Lippert T , Borlongan CV . Prophylactic treatment of hyperbaric oxygen treatment mitigates inflammatory response via mitochondria transfer. CNS Neurosci Ther. 2019;25:815‐823. 30972972 10.1111/cns.13124 PMC6630002

[48] Zhang B , Kopper TJ , Liu X , Cui Z , Van Lanen SG , Gensel JC . Macrolide derivatives reduce proinflammatory macrophage activation and macrophage mediated neurotoxicity. CNS Neurosci Ther. 2019;25:591‐600. 30677254 10.1111/cns.13092 PMC6488883

[49] Benedek G , Meza‐Romero R , Andrew S et al Partial MHC class II constructs inhibit MIF/CD74 binding and downstream effects. Eur J Immunol. 2013;43:1309‐1321. 23576302 10.1002/eji.201243162 PMC3788583

[50] Yang L , Kong Y , Ren H et al Upregulation of CD74 and its potential association with disease severity in subjects with ischemic stroke. Neurochem Int. 2017;107:148‐155. 27884769 10.1016/j.neuint.2016.11.007 PMC5438911

